# Cardiovascular magnetic resonance for evaluation of cardiac involvement in COVID-19: recommendations by the Society for Cardiovascular Magnetic Resonance

**DOI:** 10.1186/s12968-023-00933-0

**Published:** 2023-03-27

**Authors:** Vanessa M. Ferreira, Sven Plein, Timothy C. Wong, Qian Tao, Zahra Raisi-Estabragh, Supriya S. Jain, Yuchi Han, Vineeta Ojha, David A. Bluemke, Kate Hanneman, Jonathan Weinsaft, Mahesh K. Vidula, Ntobeko A. B. Ntusi, Jeanette Schulz-Menger, Jiwon Kim

**Affiliations:** 1 University of Oxford Centre for Clinical Magnetic Resonance Research (OCMR), Oxford British Heart Foundation Centre of Research Excellence, The National Institute for Health Research Oxford Biomedical Research Centre at the Oxford University Hospitals NHS Foundation Trust, Division of Cardiovascular Medicine, Radcliffe Department of Medicine, University of Oxford, Oxford, UK; 2 Department of Biomedical Imaging Science, University of Leeds, Leeds, UK; 3 Division of Cardiology, Department of Medicine, University of Pittsburgh School of Medicine, Pittsburgh, USA; 4 Department of Imaging Physics, Delft University of Technology, Delft, The Netherlands; 5 William Harvey Research Institute, NIHR Barts Biomedical Research Centre, Queen Mary University of London, Charterhouse Square, London, EC1M 6BQ UK; 6 Division of Pediatric Cardiology, Department of Pediatrics, Maria Fareri Children’s Hospital at Westchester Medical Center, New York Medical College, New York, USA; 7 Cardiovascular Medicine, Wexner Medical Center, The Ohio State University, Columbus, USA; 8 Department of Cardiovascular Radiology and Endovascular Interventions, All India Institute of Medical Sciences, New Delhi, India; 9 Department of Radiology, University of Wisconsin School of Medicine and Public Health, Madison, USA; 10 Department of Medical Imaging, Toronto General Hospital, University of Toronto, Toronto, Canada; 11 Department of Medicine, Division of Cardiology, Weill Cornell Medicine/New York Presbyterian Hospital, Weill Cornell Medical College, New York, USA; 12 Division of Cardiovascular Medicine, University of Pennsylvania, Philadelphia, USA; 13 Division of Cardiology, Department of Medicine, University of Cape Town and Groote Schuur Hospital; Cape Heart Institute, University of Cape Town, South African Medical Research Council Extramural Unit On Intersection of Noncommunicable Diseases and Infectious Diseases, Cape Town, South Africa; 14 Working Group on Cardiovascular Magnetic Resonance, Experimental and Clinical Research Center, a joint cooperation between Charité and MDC, Charité University Medicine, Berlin, Germany; 15 Department of Cardiology and Nephrology, Helios Hospital Berlin-Buch, Berlin, Germany

**Keywords:** Cardiovascular magnetic resonance, COVID-19, SARS-CoV-2, Cardiac complications, Myocarditis, Myocardial infarction, Microinfarctions, Thrombotic complications, Multisystem inflammatory syndrome, Diagnostic criteria

## Abstract

Coronavirus disease 2019 (COVID-19) is an ongoing global pandemic that has affected nearly 600 million people to date across the world. While COVID-19 is primarily a respiratory illness, cardiac injury is also known to occur. Cardiovascular magnetic resonance (CMR) imaging is uniquely capable of characterizing myocardial tissue properties in-vivo, enabling insights into the pattern and degree of cardiac injury. The reported prevalence of myocardial involvement identified by CMR in the context of COVID-19 infection among previously hospitalized patients ranges from 26 to 60%. Variations in the reported prevalence of myocardial involvement may result from differing patient populations (e.g. differences in severity of illness) and the varying intervals between acute infection and CMR evaluation. Standardized methodologies in image acquisition, analysis, interpretation, and reporting of CMR abnormalities across would likely improve concordance between studies. This consensus document by the Society for Cardiovascular Magnetic Resonance (SCMR) provides recommendations on CMR imaging and reporting metrics towards the goal of improved standardization and uniform data acquisition and analytic approaches when performing CMR in patients with COVID-19 infection.

**Supplementary Information:**

The online version contains supplementary material available at 10.1186/s12968-023-00933-0.

## Background

**Fig. 1:**
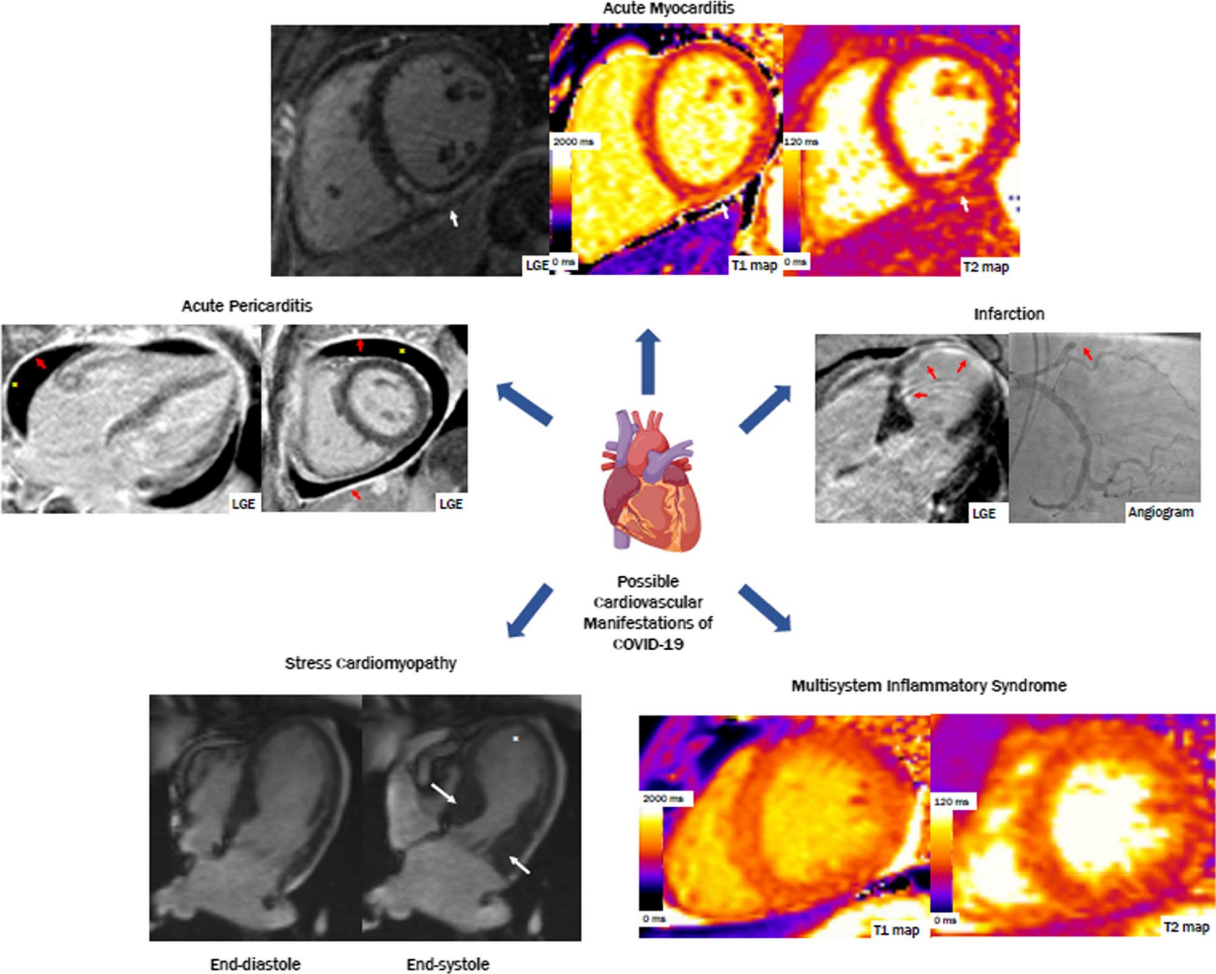
Cardiovascular manifestations of COVID-19 on cardiovascular magnetic resonance (CMR). *Clockwise from the top*: (1) A patient diagnosed with acute myocarditis, found to have midmyocardial late gadolinium enhancement (LGE) in the inferior and inferoseptal walls, with increased T2 relaxation times in the inferior wall (white arrows). (2) A patient with subendocardial LGE in the mid to distal septum and apex, found to have an occlusion in the mid left anterior descending artery on coronary angiography (red arrows). (3) Globally increased native T1 and T2 relaxation times in a patient with multisystem inflammatory syndrome. (4) A patient diagnosed with stress cardiomyopathy, with thickening of the basal segments (white arrows) and akinesis of apex (asterisk) seen on cine imaging. (5) A patient diagnosed with acute pericarditis, found to have diffuse LGE in the pericardium (red arrows) and a pericardial effusion (asterisk)

The coronavirus disease 2019 (COVID-19) pandemic is a major cause of morbidity and death worldwide. Individuals with pre-existing cardiovascular disease are at increased risk of severe illness and death in association with COVID-19 [1, 2]. Furthermore, growing evidence has highlighted COVID-19 as a multisystem disease, with an array of potential cardiovascular manifestations in the acute and post-acute phases of the illness [3]. Multiple studies report evidence of ischemic and non-ischemic myocardial injury [4–7], as well as myocardial and immune response, as part of a systemic inflammatory response in the context of acute COVID-19. Ischemic injury may relate to typical acute plaque rupture and other etiologies, such as myocardial ischemia precipitated by critical illness. However, a pro-thrombotic state and coronary vasculitis associated with COVID-19 have also been observed [8–11]. Non-ischemic etiologies of myocardial injury include myocarditis [12] and less often, stress-induced (Takotsubo) cardiomyopathy [13], both of which can manifest as acute heart failure [14] (Fig. 1). In children, while acute COVID symptoms are generally mild, a multisystem inflammatory syndrome in children (MIS-C) assumed to be a delayed hyperimmune response to SARS-COV-2 infection/exposure has been reported [15]. Cardiovascular manifestations in MIS-C can range from vasodilatory or cardiogenic shock, acute heart failure, myocarditis and/or coronary artery involvement akin to Kawasaki disease [16–18].

The long-term cardiovascular manifestations of SARS-COV-2 infection remain unknown. Several studies report protracted non-ischemic myocardial injury and/or ongoing myocarditis after apparent recovery from the acute phase of COVID-19 [19–22]. However, the longer-term significance of such observations is uncertain. Due to a high prevalence of cardiovascular disease in the general population, studies of COVID-19 patients must include appropriate control groups to improve reliability and clinical interpretation of their studies [23–25]. CMR has also been used to evaluate rare reports of myocardial injury associated with COVID-19 vaccination, particularly in male adolescents and young adults [26–30].

The clinical complexity of patients with cardiovascular involvement in the setting of COVID-19 presents unique challenges in diagnosis, clinical management, and longer-term risk stratification to optimize clinical outcomes. Cardiovascular magnetic resonance (CMR) imaging has become a leading imaging modality to detect both acute and long-term cardiovascular sequelae of COVID-19 infection due to its unique capability of detecting myocardial injury and characterizing myocardial tissue properties in-vivo. The number of reports of myocardial involvement in COVID-19 using CMR is rapidly increasing. However, comparisons between studies are hindered by variation in methodology used in acquisition and analysis methods. Previous reports by the Society for Cardiovascular Magnetic Resonance (SCMR) have provided guidance on the use of CMR during and after COVID-19 infection [31–33]. This consensus document focuses on recommendations on CMR imaging and reporting metrics, toward improved standardization, uniform data acquisition, and analytic approaches for assessing cardiac involvement in COVID-19. In accordance with SCMR guidance [34], the writing panel comprised of experts with a broad range of expertise in CMR and COVID-19 related cardiovascular manifestations and a wide geographical and subspecialty background. The panel reviewed existing literature and in accordance with available scientific evidence developed consensus recommendations for clinical CMR practice. These recommendations were then further modified following external review and approved by final consensus of the writing panel.

## Histopathological cardiac findings in COVID-19

Histopathological findings of the heart in patients with COVID-19 may advance our understanding of the underlying pathophysiology of cardiac involvement in this disease. It has been postulated that both systemic inflammatory response as well as direct organ damage by infiltration of SARS-CoV-2 virus are the putative mechanisms for myocardial injury in COVID-19. However, to date, there is little evidence supporting direct damage to cardiomyocytes due to virus-mediated lysis and the virus has been detected in the myocytes in only a few cases [35]. Although SARS-CoV-2 mRNA has been detected in myocardium in 25–50% of COVID-19 patients during autopsy, it is predominantly found within the pericytes and in the subendothelium rather than myocytes [36]. Other possible mechanisms for myocardial injury in this disease include cytokine storm, microvascular angiopathy, endothelial dysfunction and a hypercoagulable state which causes coronary arterial thrombosis [37].

A systematic pathological analysis of 40 hearts from an autopsy series of hospitalized patients who died of COVID-19 showed that the most common pathological cause of myocyte necrosis was microthrombi or small focal areas of myocardial necrosis [38]. Overall, 35% (14/40) had evidence of myocyte necrosis, predominantly of the left ventricle (LV), with no significant difference in the incidence of severe coronary artery disease (CAD) between those with and without necrosis. Of those with myocyte necrosis, 21% showed acute myocardial infarction (≥ 1 cm^2^ area of necrosis) whereas 79% showed small areas of focal myocyte necrosis (> 20 necrotic myocytes with an area of ≥ 0.05 mm^2^ but < 1 cm^2^). Further, 79% (11/14) showed cardiac thrombi; 14% (2/14) had epicardial coronary artery thrombi, whereas 64% (9/14) had microthrombi in myocardial capillaries, arterioles, and small muscular arteries. Interestingly, microthrombi from COVID-19-positive autopsy cases were different in composition from intramyocardial thromboemboli from COVID-19-negative subjects, and from coronary thrombi retrieved from COVID-19-positive and -negative patients with ST-segment–elevation myocardial infarction.

A systematic review of the post-mortem pathological findings in COVID-19, the major microscopic findings were myocardial necrosis, interstitial macrophages, lymphocytic infiltration of the myocardium and thrombosis of coronary microvasculature [39]. On immunohistochemistry, the myocardium demonstrates inflammation with infiltration of CD68 + macrophages as well as CD3 + , CD4 + and CD8 + cytotoxic lymphocytes [39]. These findings demonstrate that COVID-19 leads to an inflammatory and a prothrombotic state in the myocardium. Presence of CD3 + lymphocytes is consistent with the fact that cellular immunity plays a key role in the host response mounted during COVID-19 infection. Another review of 277 cardiac autopsies across 22 studies suggested that classical myocarditis (confluent myocyte necrosis or diffuse lymphocytic infiltration) was identified only in 7.2% with the prevalence of non-myocarditis inflammatory infiltrate, single-cell ischemia and acute myocardial infarction being 12.6%, 13.7% and 4.7%, respectively [40]. As per the current evidence, unlike other forms of viral myocarditis, fulminant myocarditis as a cause of death is rare in COVID-19 and non-specific cardiac inflammation is more common [35, 36].

### Cardiovascular imaging in the evaluation of patients with COVID-19

**Fig. 2:**
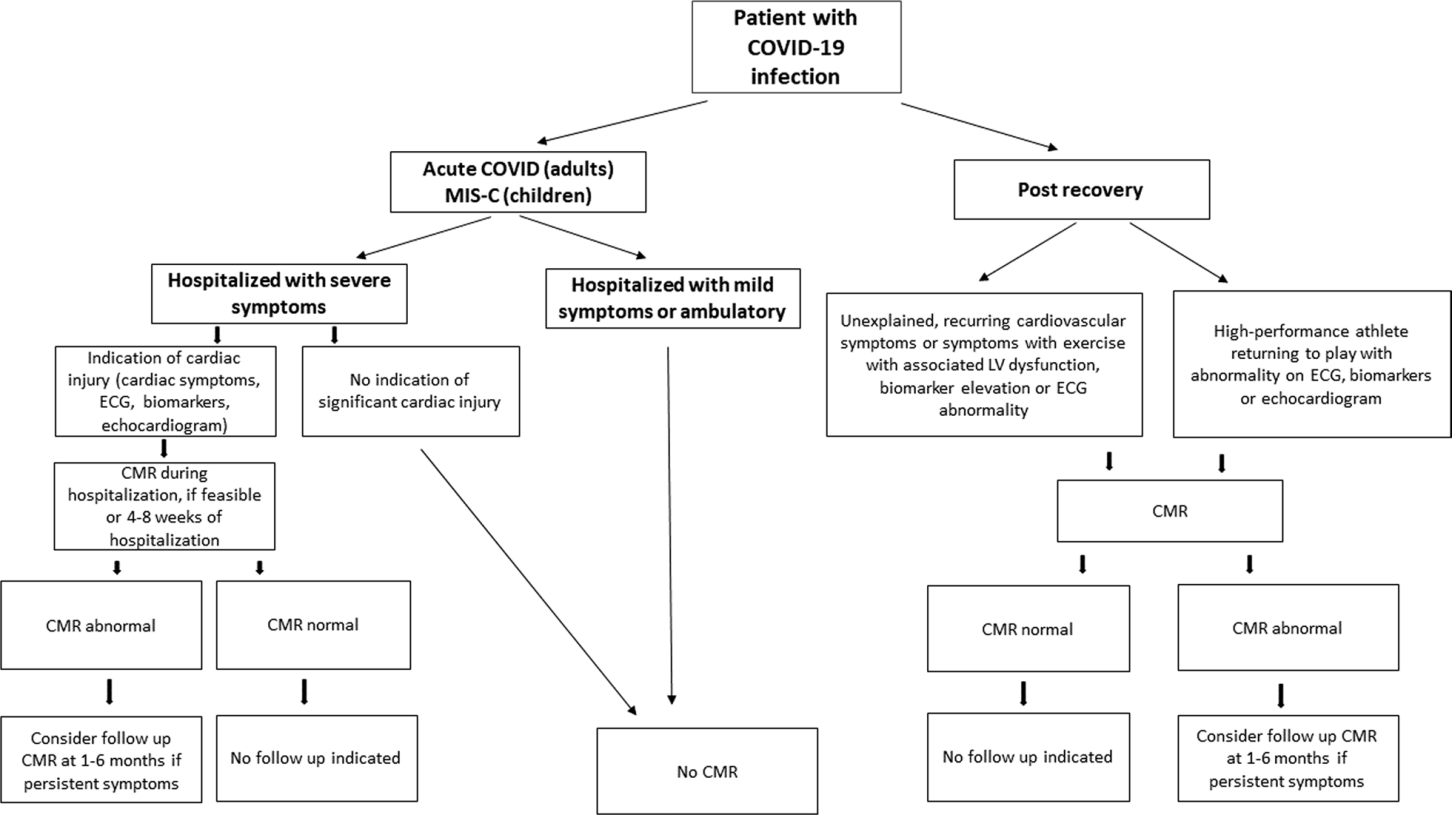
Recommendations for the use of CMR in COVID-19. As shown, CMR may be considered among patients with severe COVID-19 infection and clinical evidence of myocardial injury. Among convalescent patients with COVID-19 infection, CMR is of highest utility among patients with abnormal echocardiogram, electrocardiogram (ECG), and biomarkers as well as ongoing cardiopulmonary symptoms. *MIS-C* multisystem inflammatory syndrome in children

Although the reference standard for diagnosis of myocarditis is histopathology, routine endomyocardial biopsy for the diagnosis of myocarditis in the setting of COVID-19 is not currently recommended. In clinical practice, alongside cardiac troponin levels, cardiovascular imaging is key to diagnosis and clinical decision-making in patients with suspected myocarditis and other forms of cardiac injury, including after COVID-19 [35, 36]. Transthoracic echocardiography (TTE) is typically the first line cardiac imaging modality, and is highly valuable for functional assessment. However, TTE has limited capability for myocardial tissue characterization for disease classification related to COVID-19. Indeed, in a global survey of clinical TTE use in 1216 patients hospitalized with COVID-19, LV abnormalities were detected in 39% of patients; however, in most cases, a specific underlying cause was not identified [41]. Flurodeoxyglucose (FDG)-positron emission tomography (PET) findings have been described in patients with suspected myocarditis and after COVID-19 infection, although routine use would not be recommended, due to ionizing radiation exposure as well as availability [42, 43]. CMR offers both morphological and functional assessment and the ability to detect myocardial inflammation and injury with high accuracy, making it the ideal imaging modality to study cardiac involvement in COVID-19. In acute COVID-19 infection, CMR has the potential to improve diagnostic and prognostic assessment among patients with severe COVID-19 infection and clinical evidence of myocardial injury. Among convalescent patients, CMR is of highest utility among patients with ongoing cardiopulmonary symptoms and abnormal cardiovascular testing including electrocardiogram (ECG) and TTE (Fig. 2).

### CMR methods for the evaluation of patients with COVID-19

Cine CMR is the imaging reference standard for assessing cardiac structure and function; it provides high spatial and good temporal resolution with whole heart coverage, allowing precise assessment of both the left and right heart that can inform potential mechanism(s) of clinical symptoms, therapeutic responses, and potentially prognosis post COVD-19, like in other cardiac diseases [44]. Strain imaging (such as by myocardial tagging or feature tracking) can detect subclinical cardiac functional abnormalities [45, 46].

CMR uniquely offers non-invasive myocardial tissue characterization, and can detect a range of ischemic, non-ischemic, and inflammatory etiologies not accessible to other imaging modalities. Late gadolinium enhancement (LGE) highlights areas within the myocardium that have expanded interstitial space, typically in areas of focal fibrosis, as well as myocyte necrosis in the acute setting [47–49]. CMR patterns of LGE can distinguish ischemic from non-ischemic etiologies of myocardial injury, such as infarction versus myocarditis [50]. Of note, gadolinium-based media is the preferred contrast agent for LGE assessment. T2-weighted CMR images allows detection of focal and global myocardial edema that accompany acute myocarditis and infarction [51–55]. Thus T2-weighted CMR is particularly helpful in assessing acute myocardial injury and the acute myocardial response to systemic illnesses.

Parametric mapping techniques, such as T1-, T2- and extracellular volume (ECV) mapping, offer quantitative and pixel-wise characterization of the myocardial tissue. These methods have the potential to be more sensitive than LGE CMR for the detection of both acute and chronic myocardial disease [45, 56].

CMR can also assess large and small coronary vessels using CMR stress perfusion imaging [61] and perfusion mapping [62]. CMR evaluation of the pulmonary transit time can be used to detect subtle cardiac dysfunction [63]. Imaging of the pulmonary vessels and lungs at the time of CMR is also feasible, as part of the evaluation of organ involvement in COVID-19 [64, 65]. Taken together, these capabilities provide a powerful multiparametric approach by which CMR can identify both acute and chronic cardiovascular alterations in patients affected by COVID-19.

### CMR findings in COVID-19

Prior reports of the extent and degree of CMR findings in patients with COVID-19 have been heterogeneous. The literature to-date on CMR findings in patients with COVID-19 has recently been reviewed in detail elsewhere, and varying prevalence of CMR abnormalities has been reported (e.g., ranging from 26 to 60% among previously hospitalized patients) [66, 67]. The potential contributing factors include variability in study design, patient selection (e.g. disease severity, presence of pre-existing comorbidities or SARS-CoV2 subtypes), the phase of the COVID-19 illness when the CMR was performed (acute infection, during index hospitalization, or outpatient convalescence), imaging protocols, and diagnostic criteria. The inclusion of appropriate control groups was frequently omitted in early studies of patients with COVID-19 for logistical reasons. However, the inclusion of control groups is important due to the high prevalence of cardiovascular disease in the general population.

**Fig. 3:**
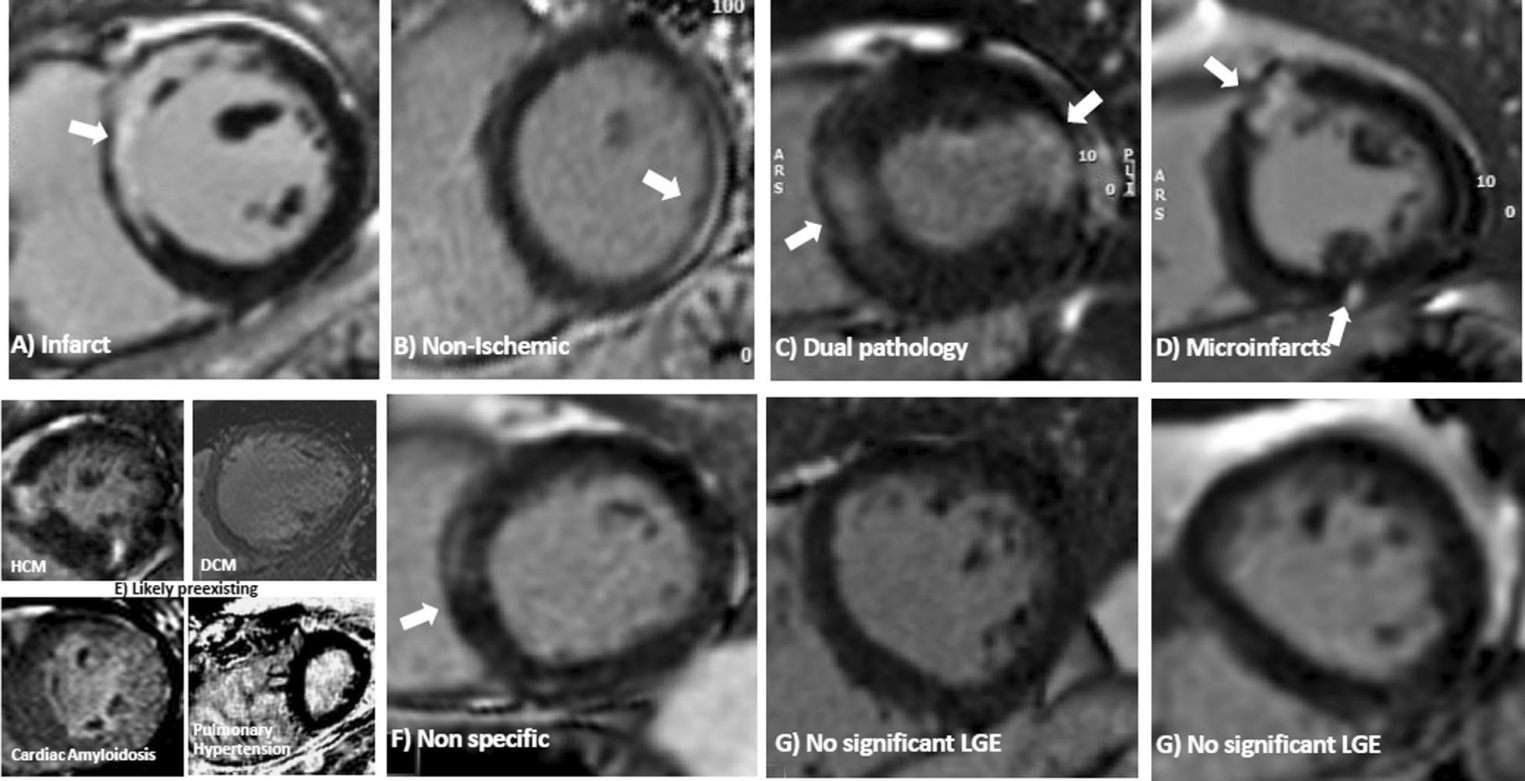
Microinfarction in COVID-19 infection. Patterns of LGE (in brackets the features of each): (**A**) Infarct (bright, subendocardial, territorial); **B** Non-ischemic (mid myocardial, less bright, more diffuse); **C** Dual pathology (both **a** and **b**); **D** Microinfarcts (bright spots—e.g. a gram or so- of LGE often but not exclusively subendocardial and potentially in more than one territory); **E** Chronic, likely pre-existent disease (only 4 cases total) and non-specific (E1: Dilated cardiomyopathy, E2: amyloidosis, E3) Non-specific (unequivocal LGE that both cannot be considered normal and has insufficient volume to assign with certainty to any other category). **F** Nonspecific (unequivocal LGE that cannot be considered normal and has insufficient volume to assign with certainty to any other category). **G** Nonsignificant LGE (minor right ventricle insertion point LGE alone; trabecular LGE alone; or septal perforator LGE alone, which can be considered normal variant) [as originally published in the COVID-HEART Study by Artico et al. [68]

COVID-HEART [68, 69] is the largest prospective, observational, longitudinal cohort CMR study to date with 342 confirmed COVID-19 and elevated troponin (COVID + /troponin +) patients across 25 hospital in the United Kingdom. Importantly, the study included two prospective control groups, comprising patients with COVID-19 and normal troponin levels (COVID + /troponin−) and patients without COVID-19 or elevated troponin but matched by age and cardiovascular co-morbidities (COVID-/comorbidity +). COVID + /troponin + patients underwent CMR within 28 days of hospital discharge and at 6-months, serum biomarkers, and genetics, amongst other investigations. Overall, COVID + /troponin + patients had a significantly two-fold higher frequency of LV dysfunction and LGE in early convalescence, compared to contemporary controls; however, the proportion with CMR imaging evidence of myocardial inflammation (6.7%) was low, and scar etiology was diverse, including a newly described pattern of probable micro-infarction (see Fig. 3). Myocardial scar, but not prior COVID-19 infection or troponin, was an independent predictor of major cardiovascular adverse event (MACE) (OR 2.25; 95% CI 1.12–4.57, p = 0.02).

This consensus document focuses on recommendations on CMR imaging and reporting metrics, toward improved standardization, uniform data acquisition and analytic approaches for assessing cardiac involvement in COVID-19. Depending on the individual case, the following CMR findings may be detected in patients who have contracted COVID-19 (see also Fig. 3):

#### Left ventricular (LV) involvement

LV dilatation and dysfunction, including impaired peak global longitudinal (GLS) and global circumferential strain (GCS) parameters, have been reported in some patients with COVID-19. This may be secondary to myocardial infarction, myocarditis, myocardial inflammation without lymphocytic myocarditis, or global myocardial injury secondary to hypoperfusion in the context of critical illness.

#### Myocardial infarction

Myocardial infarction may occur, which may be due to coronary plaque rupture in the context of acute illness, coronary occlusion promoted by up-regulation of pro-coagulant signaling pathways in COVID-19, embolization, endotheliitis, or systemic hypoperfusion. Additionally, small, punctate infarcts (microinfarctions) may be seen (Fig. 3).

#### Myocarditis and myocarditis-like CMR abnormalities

The presence of histopathologically-confirmed myocarditis in relation to SARS-CoV2 infection is thought to be low. CMR physicians are thus cautioned that clinical presentation must be considered prior to diagnoses of myocarditis related to COVID-19. Nevertheless, both typical and atypical clinical presentation may similarly be associated with non-ischemic CMR patterns in the context of COVID-19, including midwall, subepicardial, patchy, or a scattered distribution of LGE. Although a non-ischemic pattern of LGE at the right ventricular (RV) insertion points has been described, the pattern is not specific for COVID-19 [70]. Focal/global elevation of myocardial T1 and/or T2 signals have been widely reported in survivors of COVID-19 [19–21, 23, 25, 65, 70–81]. Although these signals may reflect histopathologic myocarditis (with lymphocytic infiltration and myocyte necrosis), they may also reflect upregulation of extracellular inflammation in the context of a systemic infection like COVID-19. SARS-CoV2 can involve pericytes of the myocardium independent of myocyte involvement (i.e., without myocarditis) [82]. Increased myocardial blood volume (MBV) has also been reported in patients with systemic inflammatory illnesses [83, 84]. These pathophysiologic processes may lead to acute myocardial edema that can increase myocardial T1 and T2 values, which are non-specific, and may or may not be accompanied by LGE findings. Nevertheless, the clinical significance of the observed CMR imaging abnormalities in the context of COVID-19 may be important, particularly their long-term significance in relation to symptomatology and prognosis; more longitudinal studies are required to further the understanding of these observed CMR changes.

#### Pericardial effusion and pericarditis

A pericardial effusion may be present in association with myo-pericarditis, or in the context of myocardial inflammatory response as part of a systemic illness [85].

#### Intraventricular thrombi

Both LV and RV thrombi have been described in patients with COVID-19, likely due to the prothrombotic nature of the disease [86].

#### Myocardial perfusion deficits

Inducible regional stress perfusion deficits have been described in patients who had COVID-19, some of which are thought to reflect occult pre-existing CAD [87, 88]. Global inducible perfusion deficits and LV injury may be caused by systemic hypoperfusion during moderate-severe acute COVID-19 illness. SARS-CoV-2 can directly infect the vascular endothelium, where microthrombosis and endotheliitis can result in endothelial dysfunction, microinfarctions and perfusion deficits. It is unclear at this time whether there may be long-term coronary microcirculatory abnormalities as a direct result of COVID-19.

#### Right ventricular (RV) involvement

RV dilatation and dysfunction (which may manifest as impaired RV peak GLS and GCS) may occur in patients with acute COVID-19 infection. RV dilation may be an initial compensatory adaptation to increased RV afterload and/or the augmented pulmonary circulatory requirements and parenchymal injury in context of COVID-mediated hypoxia, and may ultimately lead to increased RV wall stress and subsequent fibrosis. Adjunctive imaging of the pulmonary vasculature (via CMR angiography) and measurement of blood pool oxygenation (via T2 or susceptibility mapping approaches, and pulmonary parenchyma) may provide additive diagnostic utility in elucidating mechanism of RV injury.

### CMR findings in multisystem inflammatory syndrome in children (MIS-C) related to COVID-19

Children typically have milder acute COVID-19 symptoms, and do not have cardiac manifestations related to acute COVID-19 disease itself when compared to adults [89]. Instead, the cardiac complications seen in children are a result of a systemic delayed hyperimmune response to SARS-COV-2 (MIS-C), presenting a few weeks after the initial infection/exposure. In an initial report of the use of CMR in MIS-C from the United States, children presenting with ventricular dysfunction were studied during the acute phase of illness [90]. Although ventricular function recovered rapidly with treatment prior to discharge, there was evidence of myocardial edema, both on T2-weighted imaging and native T1 and T2 mapping, hyperemia/capillary leak on early gadolinium enhancement, and myocardial injury detected by the presence of subepicardial LGE. These findings were consistent with other reports [91–93], including one which reported ongoing ventricular dysfunction and coronary artery changes in their patients. In MIS-C, CMR detected abnormal strain in patients with global dysfunction in 35%, myocardial edema in 50%, and a subendocardial infarction in 1 patient [93].

The overall risk of myocardial involvement in children is lower than reported in the adult literature, as shown by a recent international multicenter CMR study [94], in which 82% of the sickest MIS-C patients had no evidence of CMR abnormalities. Among these patients, 18% (20/111) met the Lake Louise CMR criteria [94] for acute myocarditis. Studies evaluating early and mid-term outcomes in MIS-C have also shown that myocardial abnormalities on CMR resolve [74, 95] suggesting a favorable long-term prognosis in the pediatric population.

### CMR Findings in myocarditis following COVID-19 vaccination

Myocarditis is an established but rare adverse event following administration of mRNA-based COVID-19 vaccines. The risk of myocarditis following mRNA-based COVID-19 vaccination is highest in males between 12 and 40 years of age following administration of the second dose [28, 96–103]. The risk after the third dose is lower than following the second dose [104–106], which could be related to a longer inter-dose interval. When CMR was systematically employed to study the first sizeable pediatric cohort with vaccine-associated myocarditis in the United States, 88% of the patients fulfilled the Lake Louise myocarditis criteria [28]. A high incidence of LGE (88%) was noted in these adolescents with vaccine-associated myocarditis when compared with patients with MIS-C myocarditis [28, 94]. In adults, the incidence appears to be lower, and the extent of imaging abnormalities less severe when compared to myocarditis with acute SARS-CoV-2 infection, including having higher LV ejection fraction and less frequent involvement of the septum in vaccine-associated myocarditis [107]. Typical CMR findings include subepicardial LGE and high T2 at the basal to mid inferolateral wall.

As myocardial injury and inflammation can be present in preserved ventricular function, CMR increases diagnostic sensitivity, and should be considered in patients with suspected myocarditis following vaccination. Although most patients with myocarditis after COVID-19 vaccination have a mild initial clinical course, there are limited long-term follow-up data. In a case series of 13 adults with acute myocarditis following COVID-19 vaccination, intermediate term follow-up CMR at a median of 5 months demonstrated resolution of myocardial edema, normalization of LV function and interval decrease in LGE extent [108]. However, minimal residual LGE without edema has been documented in a proportion of patients at follow-up, likely reflecting myocardial fibrosis [108–110]. Further studies with long-term clinical and imaging follow-up are needed. Further studies are also needed to determine the risk with subsequent vaccine doses and other risk factors including prior history of myocarditis.

### Recommendations for CMR protocols for the assessment of patients with COVID-19

As for the use of CMR in evaluating other cardiac conditions, the imaging protocol should be tailored to the specific clinical question(s) and targeted towards the underlying pathophysiology. We provide a summary of common pulse sequences used for conventional and advanced myocardial characterization of these diagnostic targets and their technical considerations. Standardization of CMR protocols is important for assuring the quality, consistency, and completeness in the evaluation of cardiac involvement in patients with COVID-19. An example CMR imaging protocol is provided in Tables 1 and 2. Additional files 1 and 2 provide further details on imaging sequences and acquisition.

**Table 1:** Recommended CMR-protocols in adult patients with active/post COVID-19

Recommended CMR sequences	Answering most clinical questions
Survey	Recommended
Cine sequences: Short axis (full biventricular coverage) Long axis (HLA, VLA, LVOT) RV views (RVOT, RV 2Ch, 3Ch)	Recommended Recommended Optional
T2-weighted imaging (e.g. STIR) (myocardium/pericardium)	Optional^a^
Parametric Mapping^c^: Native T1-mapping Native T2-mapping Post-contrast T1-mapping (for ECV)	Recommended Recommended Recommended
Acquisition based myocardial strain (Tagging, DENSE, fSENC)^b^	Optional
Stress perfusion (vasodilator)^d^	Optional
Early gadolinium enhancement (EGE)^e^	Optional
Late gadolinium enhancement (LGE) Short axis full coverage and long axis views RV LGE	Recommended
Real-time cine (to assess for ventricular inter-dependence, if applicable)^f^	Optional
2D-flow (aorta and pulmonary arteries)^g^	Optional
Angiography (pulmonary vessels)^g^	Optional

*2Ch* two-chamber; *4Ch* four-chamber; *ECV* extracellular volume fraction; *HLA* horizontal long axis; *LV* left ventricle/left ventricular; *LVOT* left ventricular outflow tract; *RV* right ventricle/right ventricular; *RVOT* right ventricular outflow tract; *VLA* vertical long axis

^a^Where available, T2-mapping may circumvent some of the technical limitations of conventional T2-weighted imaging (see Additional file 1)

^b^Strain imaging may be considered if assessment for subclinical myocardial dysfunction is warranted

^c^For tissue characterization techniques, whole LV coverage will increase the diagnostic yield of detecting regions of myocardial inflammation, although this will lengthen scan time. At least 3 short-axis slices covering the LV should be obtained, recognizing that incomplete coverage will increase the potential of missing areas of myocardial inflammation

^d^In patients with cardiovascular risk factors, chest pain during COVID-19 illness may be an indication of significant underlying CAD; in these cases, it may be reasonable to include stress perfusion into the CMR protocol, to assess for signs of both obstructive CAD and myocarditis, as well as other cardiovascular changes potentially encountered in COVID-19, in a single examination

^e^EGE may be considered for thrombi detection with extension of short-axis coverage to include the atria for screening of thrombi in the atria and LV/RV

^f^Real-time cine may be considered if there is suspicion for constrictive physiology

^g^Dedicated pulmonary vascular imaging may be considered if involvement of pulmonary vasculature is suspected

### Recommendations for clinical reporting of CMR findings in COVID-19

A standardized approach to CMR image analysis, the diagnostic criteria and reporting of the CMR findings in COVID-19 is highly recommended to establish a consistent approach to the communication of the findings, including for comparison between centers, and reporting in the literature. This may facilitate a more uniform approach to the study of COVID-19 cardiac involvement using CMR as an advanced and reliable imaging modality.

#### Image analysis

CMR image quality directly affects diagnostic performance, and must be evaluated before clinical interpretation. The SCMR publishes guidelines on CMR image post-processing and interpretation [111], including the SCMR Mapping Consensus Statement (2017) [45]. A summary of these documents focusing on the myocardial tissue characterization techniques is provided here (Additional file 2).

#### Diagnostic criteria for detecting myocardial edema or inflammation on CMR in COVID-19

**Fig. 4:**
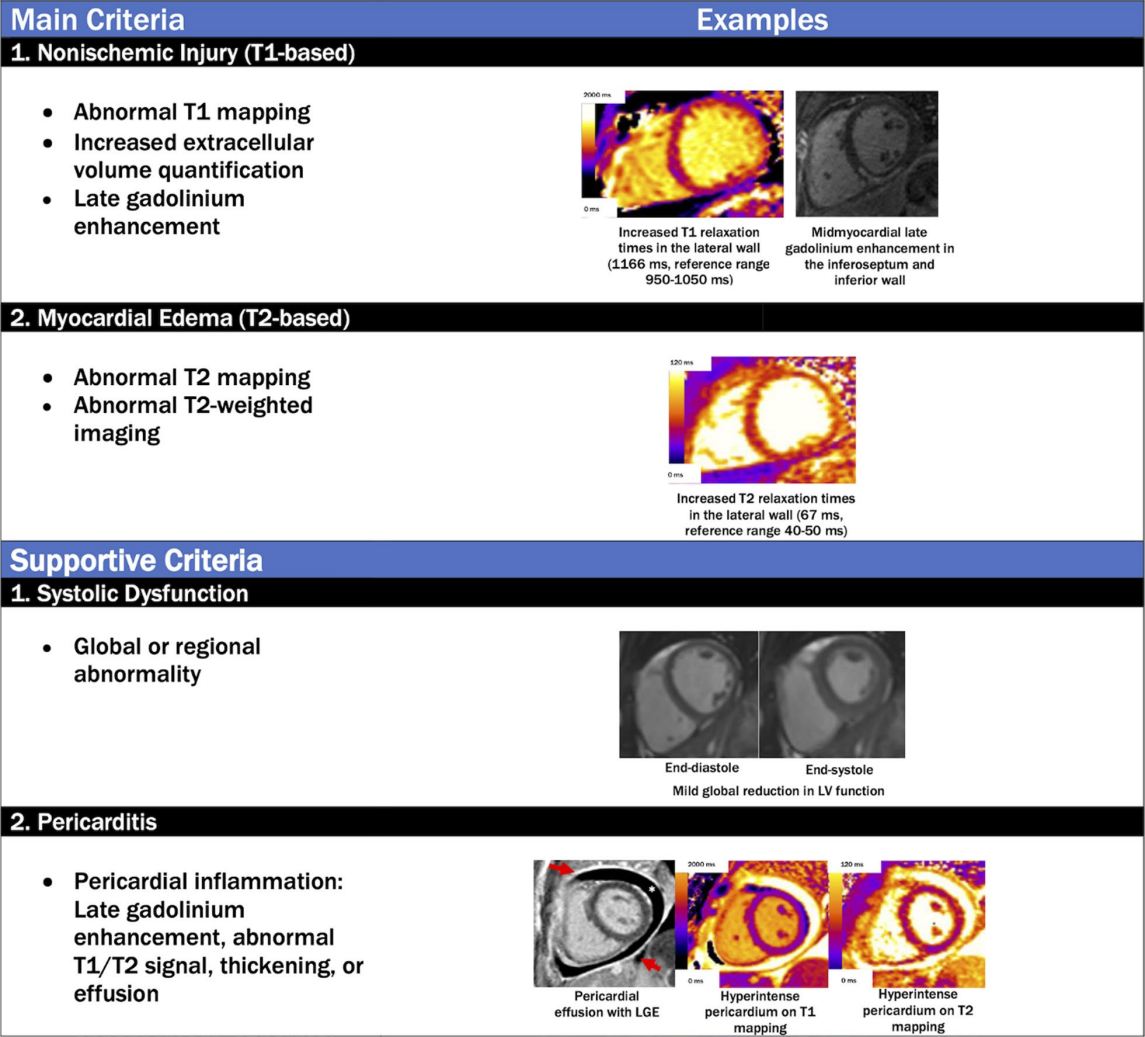
Revised Lake Louise Criteria for the diagnosis of nonischemic myocardial inflammation in patients with COVID-19. The specificity of a diagnosis of nonischemic myocardial inflammation is increased in patients meeting at least one T1-based criterion and one T2-based criterion. Supportive criteria include (1) global or regional ventricular systolic dysfunction and (2) pericardial inflammation. Red arrows indicate pericardial LGE, and the asterisk indicates the pericardial effusion

Diagnostic criteria for imaging evidence that may be consistent with non-ischemic myocardial edema and/or inflammation in COVID-19 patients can be applied using a conceptual framework provided by the “Lake Louise Criteria,” [112] most recently updated in 2018 [113] to include parametric mapping techniques (Fig. 4). It is important to note that these criteria were developed before the COVID-19 pandemic and await further validation in patients with COVID-19; nevertheless, this approach is useful for non-invasively detecting myocardial edema as a final common pathway for many forms of myocardial involvement. The specific CMR diagnostic criteria and suggested diagnostic cut-offs are summarized in Fig. 4 and Table 2. At the time when these recommendations were written, there are no pathognomonic patterns on CMR that are specific for “COVID-19 myocarditis”, but this is an evolving area in which continued accumulation of data may shed new light that will further the understanding of this disease process and its impact on the cardiovascular system. The CMR findings relating to the COVID-19 illness reported in the literature thus far are included in this document, and below.

**Table 2:** Recommended CMR-protocols in pediatric patients with COVID-19/MIS-C, Vaccine associated myocarditis

Recommended CMR sequences	Answering most clinical questions
Survey	Recommended
Cine sequences: Short axis (full biventricular coverage) Long axis (HLA, VLA, LVOT) RV views (RVOT, RV 2CH, 3Ch)	Recommended Recommended Optional
T2-weighted imaging (e.g. STIR) (myocardium/pericardium)	Recommended
Parametric Mapping: Native T1-mapping Native T2-mapping Post-contrast T1-mapping (for ECV)	Recommended (if available) Recommended (if available) Recommended (if available)
Early gadolinium enhancement (EGE)	Recommended (if available)
Late gadolinium enhancement (LGE) Short axis full coverage and long axis views	Recommended
2D-flow (aorta and pulmonary arteries)	Optional
Coronary artery (3D-navigator) imaging	Optional
Acquisition based myocardial strain	Optional
Stress perfusion	Optional
4D-flow	Optional
Angiography (pulmonary vessels)	Optional

*DENSE* displacement encoding with stimulated echoes, *ECV* extracellular volume fraction, *EGE* early gadolinium enhancement, *HLA* horizontal long axis, *LGE* late gadolinium enhancement, *LVOT* left ventricular outflow tract, *RV* right ventricle/right ventricular, *RVOT* right ventricular outflow tract, *STIR* short tau inversion recovery, *VLA* vertical long axis

#### Clinical reporting

For clinical reporting of CMR findings in patients with COVID-19, in addition to a general assessment of cardiovascular structure, function and tissue characterization, particular attention should be paid to the possible findings reported in COVID-19, as discussed earlier and in Table 3. These include: imaging signs of myopericarditis and associated pericardial effusions; small, punctate micro infarctions (Fig. 3), including the RV; associated RV dysfunction and/or dilatation; and intraventricular thrombi (within both the LV and RV). Chest pain in patients with cardiovascular risk factors during COVID-19 illness may be an indication of significant underlying CAD; in these cases, it may be reasonable to include stress perfusion into the CMR protocol, to assess for signs of both obstructive CAD and myocarditis, as well as other cardiovascular changes potentially encountered in COVID-19, in a single examination.

**Table 3:** Evaluation of CMR images and parameters for reporting cardiac findings in COVID-19

CMR parameters for reporting cardiovascular findings in COVID-19	
Ventricular structure and function	• Presence/location of global or regional LV and RV systolic dysfunction • LV & RV end-diastolic volume (LVEDV, RVEDV) • LV & RV end-systolic volume (LVESV, RVESV) • LV & RV ejection fraction (LVEF, RVEF) • LV & RV stroke volume (SV) and stroke volume index (SVI) • LV wall thicknesses • LV mass and mass index (LVMI) • Signs of RV volume or pressure overload
T2-weighted imaging	• Visual analysis: presence, extent and localization of visually apparent global or regional edema on T2-weighted imaging • Semi-quantitative analysis: global T2 SI ratio ≥ 2.0^a^ or regional^b^ high T2 SI
T1/T2 mapping	Focal/global elevation of myocardial T1 and/or T2 signals, their location and extent, which may or may not be accompanied by LGE findings or functional abnormalities • Pulse sequence (e.g. MOLLI, ShMOLLI, and relevant method version) • Field strength of CMR system • Reference normal range (mean ± SD, 2SD range) • Use only good quality parametric maps for clinical reporting • Number of slices and orientation (e.g. 3 SAx slices) • Global T1/T2 values • Segmental T1/T2 values and range may be helpful for spatial characterization • Very small regions of interest (< 20 pixels) should be avoided • The Z-score (number of SDs by which the patient findings differs from the local normal mean can help convey the degree of abnormality). A T1 or T2 value ≥ 2SD above the normal mean is generally accepted to be abnormally elevated • Clinical interpretation of whether the findings may be consistent with myocardial edema, and/or a differential diagnosis of the imaging findings within the clinical context of the referral
Edema	• Acute infarction: abnormally elevated T2 (T2-weighted or T2-mapping) in areas of infarction on LGE would support acute myocardial infarction • Non-ischemic myocardial inflammation/edema: the Updated Lake Louise Criteria (2018) recommends that one T2-based criteria (T2-weighted or T2-mapping) plus one T1-based criteria (non-ischemic LGE pattern, elevated native T1-mapping or ECV^c^) would support imaging criteria for probable non-ischemic myocardial inflammation/edema
Necrosis and fibrosis	• Presence, extent and localization of visually apparent lesions on LGE imaging • Myocardial infarctions, and if present, the location, transmurality and extent, possible coronary territory • In patients with COVID-19, small, punctate infarcts may be seen, which should be verified on perpendicular views • RV infarctions should be actively assessed for and reported • Any non-ischemic type LGE, including “myocarditis-like” type LGE patterns, such as midwall and subepicardial patterns, “scattered” or “patchy” type LGE, their extent and distribution • LGE at the RV insertion point have been described, although may have similar frequencies in individuals without COVID-19
Pericardium	• Presence, extent and localization of effusion in cine images. In general, a pericardial width > 4 mm should be regarded as abnormal • Pericardial thickness (normal ≤ 2 mm) • Signal increase in LGE, T2-weighted, T2-mapping or T1-mapping • Any hemodynamic effects or imaging evidence of constriction (such as right atrial or RV free wall collapse, ventricular inter-dependence during free-breathing cine imaging)
Thrombus	• Presence or absence of LV and RV intraventricular thrombi • Presence of thrombus in the main pulmonary artery or main branches and other cardiac chambers, if visible
2D Flow of aorta and pulmonary arteries	• Forward, backward and net flow in the ascending aorta and main pulmonary artery • Can be used to calculate mitral and tricuspid regurgitant volume and fraction along with LV & RV stroke volumes if needed • Evaluation of pulmonary emboli and lung opacities
Perfusion deficits	• Regional perfusion deficits may suggest underlying obstructive CAD • Global inducible perfusion deficits (based on quantitative analysis of myocardial blood flow) may result from systemic hypoperfusion, microvascular dysfunction from microthrombosis or endotheliitis

^a^Published or local normal values should be used; degree of LV coverage should be reported

^b^“Regional” refers to an area of at least 10 contiguous pixels

^c^Native T1 and ECV are also sensitive to, although not specific for, acute myocardial inflammation and edema, because these parameters are also sensitive to detecting chronic changes, such as in areas of focal and diffuse myocardial fibrosis

CAD coronary artery disease, SI signal intensity. Other abbreviations as in Table 1

## Conclusion

Existing published work documents a heterogenous spectrum of COVID-19 related cardiovascular manifestations depending on COVID-19 disease severity and individual patient characteristics. Multiparametric CMR allows a safe and non-invasive assessment of cardiac structure, function and, importantly, myocardial tissue characterization in COVID-19 patients. As such, CMR permits elucidation of specific diagnoses, including myocardial edema, myocardial infarction and microinfarctions, myo-pericarditis, ischemia, fibrosis, intracavitary thrombi, and non-ischemic cardiac dysfunction. Furthermore, the high reproducibility of CMR permits reliable longitudinal tracking of any observed cardiovascular changes, response to potential therapy, and association with clinical outcomes. This SCMR consensus document provides guidance on the acquisition, interpretation, and analysis of CMR images in the context of COVID-19 infection, to improve standardization of methods globally. Further research is needed to determine the biological basis of the CMR abnormalities that are observed, to enable greater understanding of underlying disease mechanisms, as well as their clinical significance with regards to function, quality of life, and long-term cardiovascular risk.

## Supplementary Information

**Additional file 1.**CMR Imaging Protocol—details on imaging sequences and acquisition.

**Additional file 2.** Clinical Reporting of CMR findings in COVID-19—Image analysis.

## Data Availability

Not applicable.

## Abbreviations

bSSFP: Balanced steady-state free precession
CAD: Coronary artery disease
CMR: Cardiovascular magnetic resonance
COVID-19: Coronavirus disease 2019
ECG: Electrocardiogram
ECV: Extracellular volume fraction
FoV: Field of view
GCS: Global circumferential strain
GLS: Global longitudinal strain
LGE: Late gadolinium enhancement
LV: Left ventricle/left ventricular
MACE: Major adverse cardiovascular events
MBV: Myocardial blood volume
MIS-C: Multisystem inflammatory syndrome in children
MOCO: Motion correction
ROI: Region of interest
RV: Right ventricle/right ventricular
SCMR: Society for Cardiovascular Magnetic Resonance
SD: Standard deviation
SI: Signal intensity
T1: Spin–lattice or longitudinal relaxation time
T2: Spin–spin or transverse relaxation time
TTE: Transthoracic echocardiography
