# Supplementary material for: Cardiovascular magnetic resonance for evaluation of cardiac involvement in COVID-19: recommendations by the Society for Cardiovascular Magnetic Resonance
Source: J Cardiovasc Magn Reson. 2023 Mar 27;25:21. doi: 10.1186/s12968-023-00933-0 (PMC10041524; doi:10.1186/s12968-023-00933-0)
Supplement: Supplementary file 1 — Additional file 1.CMR Imaging Protocol—details on imaging sequences and acquisition. [file 12968_2023_933_MOESM1_ESM.docx]

**ADDITIONAL FILE 1**

**CMR Imaging Protocol – details on imaging sequences and acquisition**

*T2-weighted imaging*

Edematous myocardium resulting from inflammation (e.g. myocarditis) or early necrosis (e.g. acute myocardial infarction) may be visualized using T2-weighted imaging. Conventionally, two general techniques are in common use: dark-blood T2-weighted imaging (such as turbo spin-echo following double inversion recovery to suppress blood signal or triple inversion recovery to suppress blood and fat; and bright-blood T2-weighted imaging (i.e. “T2-prep” combined with balanced steady state free precession). Artifacts commonly encountered in these T2-weighted images may both mimic and/or obscure actual features.

In dark-blood T2-weighted imaging, a slice thickness of 8-10 mm is recommended to maximize the signal-to-noise ratio (SNR); slice thicknesses that are too thin may result in degraded image quality with insufficient myocardial signal and non-diagnostic images. The body coil (without surface coils) may be used to provide a uniform signal across the field of view (FoV), especially for comparing T2 signals from the myocardium to skeletal muscle. If surface coils are used, signal intensity correction may be applied, to minimize signal variation across the myocardium and FoV. Signal drop-out can be observed in the lateral wall, especially in the short-axis basal slices, which may create a false appearance of relatively “bright” anterior or septal walls. Slow-flow blood within the cardiac chambers may not suppress adequately, and artifactual bright subendocardial signal are common, due to stagnant blood or blood flow that is not perpendicularly directed to the imaging plane. Imaging in arrhythmia can be especially challenging, particularly for dark-blood T2 sequences.

*Late gadolinium enhancement*

LGE CMR is performed using multi-shot (or segmented) inversion recovery fast gradient echo (IR-GRE) and/or single-shot bSSFP; both may be performed with or without phase sensitive inversion recovery reconstruction. Small areas of LGE in subendocardial and subepicardial locations may be difficult to differentiate from adjacent blood pool and pericardium, respectively. Suspected findings should be confirmed on 2 adjacent imaging planes, or 2 different imaging axes. Myocardial thickness can be compared on cine images (especially if acquired prior to contrast administration) to determine whether the enhancement occurs in the myocardium or blood pool. Black blood LGE techniques are increasingly available to improve imaging of the blood-subendocardium interface. Microinfarctions may be more readily apparent using high resolution navigator gating approaches. Right ventricular (RV) infarction may be better highlighted using tailored inversion times for RV myocardium, as well as high spatial resolution imaging for detection of small infarcts. Epicardial and/or pericardial fat may mimic true epicardial LGE of the myocardium, and adjudication of such findings may be assisted by comparison with T2-weighted imaging (with and without fat suppression), or the inclusion of T1 weighted imaging (including T1-mapping) prior to contrast administration. Enhancement of the RV-LV insertion point can be seen in otherwise normal individuals, and has also been reported in patients with COVID-19; these should be distinguished from partial volume effects where blood pool from the insertion of RV septomarginal trabeculae is erroneously identified as abnormal enhancement. Other common sources of apparent LGE should be recognized before ascribing to COVID-19; these include: prominent septal perforator coronary artery branches of the left anterior descending coronary artery, commonly seen in the basal anterior septum, may appear as midwall or subepicardial enhancement in this area; myocardial crypts or clefts, typically in the basal inferior wall, may appear as subendocardial infarctions on LGE images. Review of movie cine imaging, including in long-axis planes, may help to accurately adjudicate these potentially confounding features.

*Parametric T1 and T2 mapping tissue characterization*

The SCMR provides periodic updates on recommended imaging protocols, and these should be consulted using the latest version available. The following incorporates recommendations from the SCMR Standardized cardiovascular magnetic resonance imaging (CMR) protocols: 2020 update^114^ and the SCMR Mapping Consensus Statement (2017).^45^

*T1-mapping*

1. Native T1 mapping is performed in the absence of contrast or stress agents, at rest.
2. Look-Locker imaging (modified Look-Locker Inversion recovery (MOLLI) or shortened MOLLI (ShMOLLI)^115^ or equivalent should be used.^114^
3. Slice thickness: 6–8 mm, in-plane resolution ~1.6–2.0 mm
4. Volume-selective B_0_ shimming focused on the heart is highly recommended at 1.5 T, and essential at 3 T. B_1_ (radiofrequency) volume shimming is recommended at 3 T.
5. Diastolic acquisition is recommended if there is regular normal heart rhythm; in patients with tachyarrhythmia, specific sequences designed for higher heart rates can be useful. In patients with atrial fibrillation, image acquisition should be repeated to allow for averaging of the results. Systolic readout has been shown to produce robust T1 maps in tachyarrhythmias^116^ but may require specific normal values.
6. Image quality should be reviewed during acquisition (e.g. by monitoring sequence sounds and electrocardiographic (ECG) gating), and by looking at source images, error maps, and other quality control maps. Suboptimal scans should be repeated.
7. If in-plane motion correction (MOCO) is used, this needs to be carefully checked for introduction of any new artefacts and is not a replacement for breath-holding in non-navigated techniques. MOCO may not robustly compensation for extreme respiratory motion, through plane motion, or ECG gating difficulties.
8. The number and orientation of slices obtained will depend upon the indication. At least one short-axis map should always be obtained. Whole-heart coverage will provide more spatial-information and diagnostic yield but should be balanced against increased scan time and patient fatigue.
9. For ECV measurements, T1 mapping should be performed prior to contrast and at least 1 time point between 10- and 30-min post contrast bolus. An extracellular gadolinium-based contrast agent with non-protein bound distribution (0.1 – 0.2 mmol/kg) should be used for the assessment of ECV. Post-contrast T1-maps should match native T1-maps in slice position and other prescribed imaging parameters (such as FoV and cardiac phase).
10. The hematocrit should be measured just before the scan and, ideally within 24 h of imaging, for the accurate ECV measurement.

*T2-mapping*

1. Multiple alternatives exist, such as T2-prepared single-shot bSSFP sequence acquired with different T2 prep time, gradient spin echo (GraSE) or FSE-based pulse sequences.
2. Slice thickness: 6-8 mm, in plane-resolution ~ 1.6–2.0 mm
3. Diastolic acquisition is recommended
4. T2-mapping sequences may exhibit heart-rate dependencies, and may require adjustments when imaging during tachycardia
5. The number and orientation of slices obtained will depend upon the indication. Short-axis maps should always be obtained.
6. Source images should be checked for motion/artefact, including in MOCO images, and imaging repeated if this occurs.

Normal reference ranges

Quantitative methods, such as T1- and T2-mapping, require correct implementation on the scanner and quality control to ensure reliable measurements. ^45, 117^ Given the multitude of technical factors that can influence their accuracy, it is essential to standardize or harmonize methodologies, especially for inter-center comparisons, as well as to keep the methods stable for longitudinal comparisons.^45^ In the absence of universally standardized parametric mapping methods, individual centers should use validated sequences, and establish local normal ranges that are benchmarked against known norms for the methods used.^45, 118^

Per the SCMR Mapping Consensus statement (2017), local normal ranges should be based on a minimum of 15 healthy controls for detecting pathologies with large signal deviations from norm, but may require up to 50 controls for detecting pathologies with small signal changes (such as diffuse fibrosis). This may involve scanning healthy volunteers using these sequences, obtaining coverage of the LV for its intended use in the patient population (e.g. 1 mid-ventricular slice, 3 short-axis slices, whole heart coverage, etc.), and analyzing the images to provide global and/or segmental norms.

Normal ranges of myocardial native T1, T2 and ECV methods may exhibit age and sex dependencies, in addition to methods of image analysis; care should be taken with regard these factors when reporting and classifying disease and norm.

In centers where establishing local normal ranges may be challenging, alternate solutions are possible, such as using well-established sequences with quality assurance approaches that the method has been implemented correctly at the local scanner,^117^ and once quality assessment (QA) has passed, sharing of normal reference range databases between centers using the same sequences may be feasible. QA should also be conducted after scanner or software upgrades, to ensure no errors have been introduced into the quantitative parametric sequences previously verified to be implemented correctly.^45, 117^
