# Supplementary material for: Cardiovascular magnetic resonance for evaluation of cardiac involvement in COVID-19: recommendations by the Society for Cardiovascular Magnetic Resonance
Source: J Cardiovasc Magn Reson. 2023 Mar 27;25:21. doi: 10.1186/s12968-023-00933-0 (PMC10041524; doi:10.1186/s12968-023-00933-0)
Supplement: Supplementary file 2 — Additional file 2. Clinical Reporting of CMR findings in COVID-19—Image analysis. [file 12968_2023_933_MOESM2_ESM.docx]

**ADDITIONAL FILE 2**

**Clinical Reporting of CMR findings in COVID-19 - Image analysis**

*T2-weighted imaging*

Visual analysis of T2-weighted images aims to identify myocardial regions with high signal intensity (SI), which provides supportive imaging evidence of myocardial edema. These may be regional (when compared to unaffected areas within the LV myocardium) or global (when compared to a reference region-of-interest (ROI) such as in skeletal muscle); in some cases, patterns of injury (e.g., subendocardial, midwall, subepicardial) may be evident. Adjustment of the image contrast and brightness will aid visual identification of areas of relatively high T2 SI. Verification of the suspected area of high T2 SI in perpendicular views or at least two consecutive slices will increase diagnostic confidence. Areas of high T2 SI may be compared to other imaging sequences, such as cine and LGE, to corroborate findings in areas of abnormality, but edema may occur without functional consequences or LGE. In dark-blood triple-inversion recovery spin echo images (e.g., STIR, TIRM), pitfalls to avoid include relatively low SI in the posterior (lateral) wall occasionally seen (especially in basal slices with through-plane motion), and failure to suppress slow-flow blood in the LV cavity along the subendocardium (particularly in the apex.

*Late gadolinium imaging*

Visual analysis of LGE images is typically used for most clinical indications. Image quality should be assured by prescribing an appropriate inversion time (TI) to null normal myocardium to highlight the abnormal areas; inappropriate TI may result in false positive or false negative findings. Cardiac or breathing motion may significantly impact on the diagnostic utility of LGE images; MOCO and free-breathing options may sometimes circumvent these technical difficulties. LGE presence should be further characterized according to the pattern, extent, and spatial distribution. Verification of LGE-positive areas in perpendicular views, after changing the direction of readout in the same plane (i.e., “swapping”), or reproducing the same lesion using a different LGE sequence, will increase diagnostic confidence. Pitfalls to note include bright artefacts due to motion or inappropriate TI; small subendocardial LGE may be difficult to differentiate from the bright blood pool, in which case waiting for washout of the gadolinium based contrast agent from the intracavitary blood pool and repeating imaging or using dark-blood/grey-blood LGE sequences, may be helpful. Semi-quantitative analyses are usually reserved for research purposes, and further details on different approaches are available elsewhere.^111^

*Parametric (T1/T2/ECV) mapping*

For parametric mapping, the SCMR provides guidelines on image analysis in the image post-processing and interpretation^111^ and mapping consensus statements^45^. Parametric maps are directly quantitative pixel-wise images, and thus, to ensure robust image quality and diagnostic accuracy, it is imperative that the entire pipeline is quality controlled; this includes method installation, image acquisition, and assessment of the image quality of the resultant parametric maps, before using for clinical diagnosis. Parametric maps may be corrupted by motion (cardiac, respiratory or body), mistrigerring, susceptibility and other artefacts. The image quality of parametric maps may be assessed by examining the raw T1/T2-weighted images, the T1/T2 curve-fit using appropriately validated or commercially-available software, and any accompanying quality control maps. Quality control maps may be presented in R-squared (R^2^) maps (the square of Pearson correlation, i.e. coefficient of explained variance) or residual maps (which estimate the T1 or T2 standard deviation); these can provide a visual display of the quality of the parametric map before using for clinical reporting.^119^

Visual analysis of parametric maps is not standardized, and various approaches are available. T1 and T2 maps may be presented on a color scale using look-up tables (LUT); ideally these should highlight areas of abnormality to the reader for easy interpretation, although diagnostic cut-offs are often required, which rely on establishment of reliable normal ranges at the minimum. Clinically-validated thresholds in specific populations and diseases may improve diagnostic ability to detect diseases, but until standardization is achieved, these may not be directly translatable between centers and methods, and MR systems.

Quantitative image analysis may be performed using dedicated software and computer-assisted post-processing. Human operators should be trained to analyze parametric maps, as parametric mapping can suffer from partial volume effects, and subjective elements can introduce variability and inaccuracies.^120^ Automated image analysis approaches using artificial intelligence methods may circumvent inter-operator variability observed in human operators, but nevertheless require quality control for reliability in clinical reporting.^121-125^ Typically, the LV myocardium is segmented using endo- and epi-cardial contours, often in short-axis views, which can then be divided into segments (e.g. American Heart Association segments), for global and segmental T1/T2/ECV values. The use of diagnostic thresholds can then help identify areas of abnormality, whether on a global or segmental level, within a specific ROI, or to obtain a percent area of abnormality on an image slice.^126^

Manual ROIs may be placed into area of interest to provide an average T1/T2 value. A septal ROI drawn only on the mid-ventricular short-axis slice is sometimes used for diseases expected to be homogenous; this approach can circumvent issues relating to partial volume or artefacts especially in a thin lateral wall, but does not maximize the use of these pixel-wise parametric maps to the full extent, particularly the spatial information and potential regional variations in diseases.^126^ Very small areas of ROIs (<20 pixels) should be avoided. Semi-quantitative approaches based on multiples of standard deviations compared to reference ROI (i.e. “remote myocardium" in ischemic heart disease) or to reference cohorts are also used. More advanced parameters for tissue characterization include measures of tissue heterogeneity, maximum-minimum pixel T1/T2 value differences, or SD measures within an ROI.^127^

ECV quantification requires a pre-contrast and a matching post-contrast T1-map, together with the hematocrit value. The paired T1-maps should match in position and other image parameters (such as field-of-view, cardiac position and phase).^45^ Image quality of the T1-maps should be assessed as discussed above with the same considerations, in addition to possible position mismatch between the pre and post-contrast T1-maps due to possible patient movement during the time elapsed between the two acquisitions. For image analysis, the LV myocardium is segmented using endo- and epicardial contours, with ROIs placed also in LV blood pool (avoiding papillary muscles and trabeculae) to provide pre- and post-contrast blood T1 values. Manual ROIs may also be placed in specific regions of interest (e.g., septum or other segments), matching on the pre- and post-contrast T1-maps, to generate region-based ECV values. The generation of ECV maps is possible if successful image co-registration is achieved between the pre- and post-contrast T1-maps using appropriate software^128^; this provides spatial information and visualization, in addition to just ECV quantification. The hematocrit value is usually obtained from blood on the same day as the CMR scan. Alternatively, non-invasive approaches including point of care devices for hematocrit assessment and derivation of “synthetic hematocrit” from the native blood T1 values have been described.^129^
